# Effects of electroacupuncture on postoperative cognitive dysfunction and its underlying mechanisms: a literature review of rodent studies

**DOI:** 10.3389/fnagi.2024.1384075

**Published:** 2024-03-26

**Authors:** Wenbo Zhao, Wei Zou

**Affiliations:** ^1^Heilongjiang University of Chinese Medicine, Harbin, China; ^2^The First Affiliated Hospital of Heilongjiang University of Chinese Medicine, Harbin, China

**Keywords:** postoperative cognitive dysfunction, electroacupuncture, animal studies, mechanisms, literature review

## Abstract

With the aging of the population, the health of the elderly has become increasingly important. Postoperative cognitive dysfunction (POCD) is a common neurological complication in elderly patients following general anesthesia or surgery. It is characterized by cognitive decline that may persist for weeks, months, or even longer. Electroacupuncture (EA), a novel therapy that combines physical nerve stimulation with acupuncture treatment from traditional Chinese medicine, holds potential as a therapeutic intervention for preventing and treating POCD, particularly in elderly patients. Although the beneficial effects of EA on POCD have been explored in preclinical and clinical studies, the reliability of EA is limited by methodological shortcomings, and the underlying mechanisms remain largely unexplored. Therefore, we have synthesized existing evidence and proposed potential biological mechanisms underlying the effects of EA on neuroinflammation, oxidative stress, autophagy, the microbiota-gut-brain axis, and epigenetic modification. This review summarizes recent advances in EA and POCD, provides a theoretical foundation, explores potential molecular mechanisms for the prevention and treatment of POCD, and offers a basis for conducting relevant clinical trials.

## 1 Introduction

Postoperative cognitive dysfunction (POCD) is a common complication affecting the central nervous system (CNS) of elderly patients. It is characterized by severe cognitive deficits, including impaired memory, attention, orientation, and abstract thinking, which may result in prolonged hospitalization, reduced independence, and higher mortality rates among this population (Yang et al., 2022). Additionally, older patients with POCD have a greater risk of developing dementia or experiencing a decline in their cognitive trajectory (Nemeth et al., 2017). Elderly patients undergoing hip fracture surgery have increased neurocognitive dysfunction during the awakening period and hospitalization (Sulaiman et al., 2014; Tan et al., 2023). Moreover, an early multicenter study investigated changes in cognitive performance after abdominal and orthopedic surgery in 1,218 elderly patients, with delayed neurocognitive recovery diagnosed in 25.8% of patients (Kong et al., 2022). Research indicates that POCD occurs in 25–40% of elderly postoperative patients, with the condition affecting up to 54% in the initial weeks following surgery, significantly compromising their quality of life and imposing a higher financial burden (Newman et al., 2001). While prevention is the best approach for POCD, involving early identification and management of perioperative risk factors, effective treatments for established POCD are still lacking (Kotekar et al., 2018). Therefore, devising potential preventive or therapeutic strategies for POCD has become a pressing need.

Acupuncture, considered a complementary and alternative treatment technique, has undergone preliminary studies in both preclinical and clinical settings. Electroacupuncture (EA), a form of acupuncture that is quantifiable, enables the control of stimulation frequency and intensity, thus eliciting varying responses in the central and peripheral nervous system during clinical studies. A systematic review of five randomized controlled trials has shown improvements in POCD with acupuncture, although the findings are often limited by biases in methodological quality (Kim et al., 2019). Furthermore, a meta-analysis focused on the use of EA for preventing POCD in older adults who have undergone hip or knee arthroplasty has highlighted that EA pretreatment can be an effective intervention for improving cognitive outcomes (Ou et al., 2021). In recent years, an increasing body of research has revealed the positive effects of EA in either preventing or addressing postoperative cognitive deficits observed in animal models. These benefits are linked to a range of mechanisms, including the reduction of neuronal death, suppression of NLRP3 inflammasomes, stimulation of new neuron growth, enhancement of endogenous Nrf2-mediated antioxidant defenses, and an increase in miR-124 expression within the hypothalamus and hippocampus.

Given that the potential mechanisms of EA for POCD are largely unexplored, we have conducted a comprehensive survey of animal model studies related to EA for POCD. Our analysis of the potential mechanisms offers insights that may help researchers better understand the progress in this field.

## 2 Study selection

**Figure 1:**
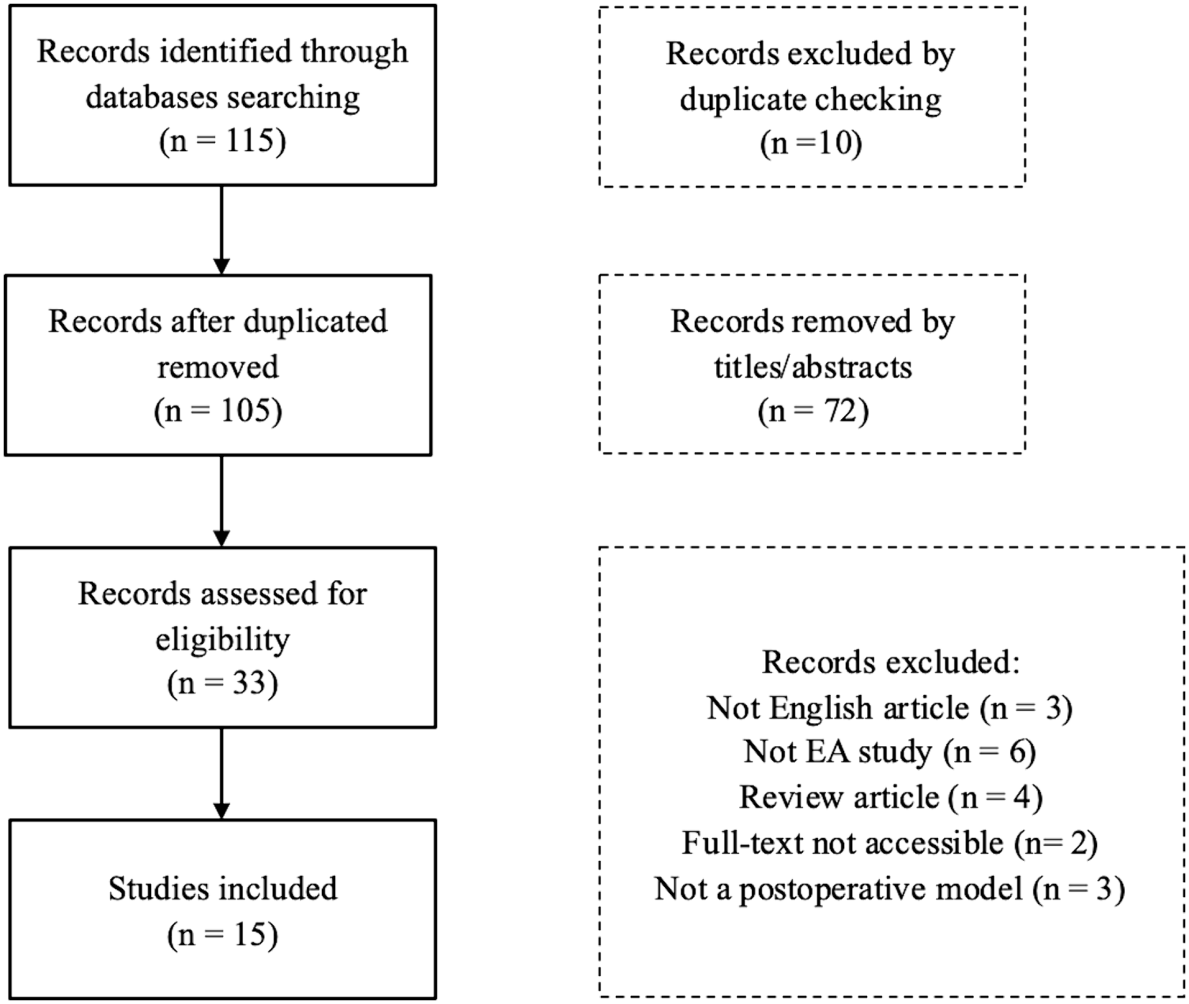
Flowchart of the study.

We conducted comprehensive searches of PubMed, Cochrane Library, Embase, Web of Science, and Scopus for literature published in English up to January 15, 2024, using a specific set of search terms: “(acupuncture OR electroacupuncture) AND (rodent OR mouse OR rat OR mice) AND (postoperative cognitive dysfunction OR surgery OR cognitive dysfunction OR postoperative).” We tailored this search strategy to suit the indexing styles of the different electronic databases. Two reviewers independently screened the titles and abstracts of the retrieved records, applying predefined inclusion criteria focused on the subjects (animal models with postoperative cognitive dysfunction), the intervention (electroacupuncture), and the outcomes (mechanisms). We identified a total of 115 records, and from these, 15 animal studies that investigated the use of EA to ameliorate POCD were selected for inclusion in our review. Figure 1 illustrates the detailed process of study selection for this review.

A total of 115 records were identified, of which 15 studies were included. One hundred studies were excluded for various reasons: 72 were excluded based on abstract and title, and 10 were excluded due to duplicate data. The remaining 18 studies were excluded due to five factors: 3 were not in English, 6 were not studies on EA, 4 were review articles, 2 were excluded because the full text could not be accessed, and 3 did not meet the criteria for the POCD model.

During the full-text assessment, multiple parameters were collected from the 15 selected studies for further analysis and comparison. Specifically, the literature was described in terms of modeling, acupuncture points, EA parameters, cognitive assessment criteria for animal models, sample selection, relevant mechanisms, and targets (Table 1). Most studies on the model of POCD utilized abdominal and hepatectomy. Moreover, two studies employed tibial fracture (Wang et al., 2021; Zhou et al., 2022), while femoral fracture (Wang M et al., 2022; Wang XF et al., 2022) and appendectomy (Zhu et al., 2023) were each used in one study. The choice of acupuncture points for EA included GV20, LI4, GV14, PC6, ST36, GV24, GB13, LI11, and the frontoparietal suture. EA parameters encompassed: current intensity from 0.2 mA to 3 mA; frequency from 2 to 100 Hz; waveforms including square wave, disperse-dense, continuous, sparse-dense; and total treatment duration from 3 to 11 days. The most commonly used criteria for cognitive function assessment were the Morris Water Maze (MWM) test, Open Field Test (OFT), and Novel Object Recognition Test (NORT). Additional assessment criteria included the Y-maze Test and Puzzle Box Test (Ho et al., 2022). Sample selection in all studies was primarily the hippocampus, though several studies also included blood and feces (Liu et al., 2019; Ho et al., 2022; Wang M et al., 2022; Wang XF et al., 2022; Zhou et al., 2022; Zhu et al., 2023). The hypothalamus (Chen L et al., 2019; Chen Y et al., 2019) and frontal cortex (Ho et al., 2022) were each investigated in one study.

**Table 1:** Effect of electroacupuncture on postoperative cognitive dysfunction and its underlying mechanisms.

Authors	Surgical model	Acupoint	EA parameter	Cognitive function assessment	Site of sample collection	Involved in pathways	Mechanism
Chen L et al. (2019); Chen Y et al. (2019)	Abdominal Rat	ST36	2 mA; 2 /15 Hz (sparse-dense); 30 min; −	–	Hypothalamus, hippocampus	miR-124/VAMP3	miR-124**△** VAMP3, IL-6, TNF-α, Microglial**▽**
Sun et al. (2022)	Hepatectomy Mice	GV20	0.5 mA; 2 Hz; 20 min; twice daily; 7 days.	MWM test	Hippocampus	NF-κB, NLRP3 inflammasome,	Microglial, IL-1β, IL-6**▽**
Wang et al. (2023)	Abdominal Mice	GV20	1 mA; 2/15 Hz (disperse-dense); 10 min; 3 days.	MWM test	Hippocampal	–	Telomerase activity, TERT protein, SOD, LC3B-II/LC3B-I, Beclin-1**△** ROS, MDA, Iba1, IL-6, TNF-α**▽**
Huang et al. (2023)	Abdominal Rat	PC6, LI4, ST36	1 mA; 2/15 Hz (sparse-dense); −; 5 days.	MWM test, NORT, OFT	Hippocampus	ERK/MAPK/ CREB	Synaptic plasticity**△**
Wang M et al. (2022) and Wang XF et al. (2022)	Femoral fracture Rat	GV24, GB13, frontoparietal suture	0.2 mA; 2/15 Hz (sparse-dense); 20 min; −	MWM test, OFT	Hippocampus, blood	–	IL-1β、IL-6, TNF-α**▽** Neuron**△**
Zhang et al. (2022)	Abdominal Rat	LI4, PC6, ST36	1 mA; 2/15 Hz (sparse-dense); −; 5 days.	MWM test, OFT	Hippocampus	Mitochondrial, Neuroapoptosis	Neuron, Ca^2+^, Bcl-2**△** ROS, Cyt c, Bax, cleaved caspase 9, cleaved caspase 3, Bax/Bcl-2**▽**
Wang et al. (2021)	Tibial fracture Rat	PC6, LI4, ST36	–; 2/15 Hz; −; −	NORT, MWM test,	Hippocampus	NF-κB, α7-nAChR	Neuron**△** Iba1, HMGB1, NF-κB (microglia), IL-6, IL-1β**▽**
Ho et al. (2022)	Laparotomy Mice	GV20, ST36	–; 1/20 Hz (sparse); 20 min; 5 days.	NORT, OFT, Y-maze Test, Puzzle Box Test	Plasma, frontal cortex, hippocampus	JAK/STAT	tau phosphorylation**△** GFAP, Iba1**▽**
Liu et al. (2017)	Hepatectomy Rat	GV20, PC6, LI4	4 mA; 2/100 Hz (continuous); 30 min; 7 days.	MWM test	Hippocampus	α7-nAChR	α7-nAChR neuron**△** TNF-α, IL-1β**▽**
Zhu et al. (2023)	Appendectomy Mice	ST36, LI11, GV20, GV14	–; 15 Hz (continuous); 30 min; 11 days.	NORT, MWM test,	Hippocampus, fecal	Gut microbiota	PDGF**△** Helibacteraceae, Actinomycetes, Clostridium sensu stricto 1, Escherichia/Shigella, GFAP, DAO, LPS, TNF-α、IL-1, IL-6**▽**
Niu et al. (2022)	Laparotomy Rat	GV14, GV20	1 mA; 3.85/6.25 Hz (sparse-dense); 30 min; 5 days.	MWM test	Hippocampus	AMPK	LC3II, Beclin-1, AMPK, p-AMPK, autophagic vesicles**▽**
Liu et al. (2019)	Hepatic lobe resection Rat	GV20, PC6, LI4	4 mA; 2/100 Hz (continuous); 30 min; 8 days.	MWM test	Hippocampus, blood	–	GFAP, S-100β, NSE, MDA**▽** BDNF, GDNF, SOD**△**
Feng et al. (2017)	Hepatectomy Rat	GV20, GV14	1 mA; 15 Hz (square); 30 min; 5 days.	MWM test	Hippocampus	–	IL-1 β、IL-6、TNF-α, HMGB-1, TLR 4/2**▽**
Zhou et al. (2022)	Tibial Fracture Rat	GV20	1 mA; 2/15 Hz; 30 min; 5 days.	MWM test	Blood, hippocampus	α 7nAChR	IL-1β, HMGB-1, TNF-α, mast cell, apoptosis**▽**
Yuan et al. (2014)	AMIR Rat	GV20, ST36	3 mA; 5 Hz; 30 min; −.	8-arm radial maze test	Hippocampus, blood	–	Iba-1, MDA, IL-6, TNF-α, TUNEL**▽** SOD**△**

MWM, Morris water maze; SOD, superoxide dismutase; ROS, reactive oxygen species; MDA, malondialdehyde; NORT, novel object recognition test; OFT, the open-field test; HMGB1, high mobility group box 1; NF-κB, nuclear factor-κB; GFAP, glial fibrillary acidic protein; S-100β, S100 calcium binding protein B; NSE, neuron-specific enolase; BDNF, brain-derived neurotrophic factor; GDNF, glial cell-derived neurotrophic factor; HMGB-1, high-mobility group box-1; IL-1β, interleukin-1β; α 7nAChR, alpha-7-nicotinic acetylcholine receptor; TNF-α, tumor necrosis factor-α; AMIR, acute myocardial ischemia–reperfusion.

## 3 Biological mechanism of electroacupuncture for postoperative cognitive dysfunction

**Figure 2:**
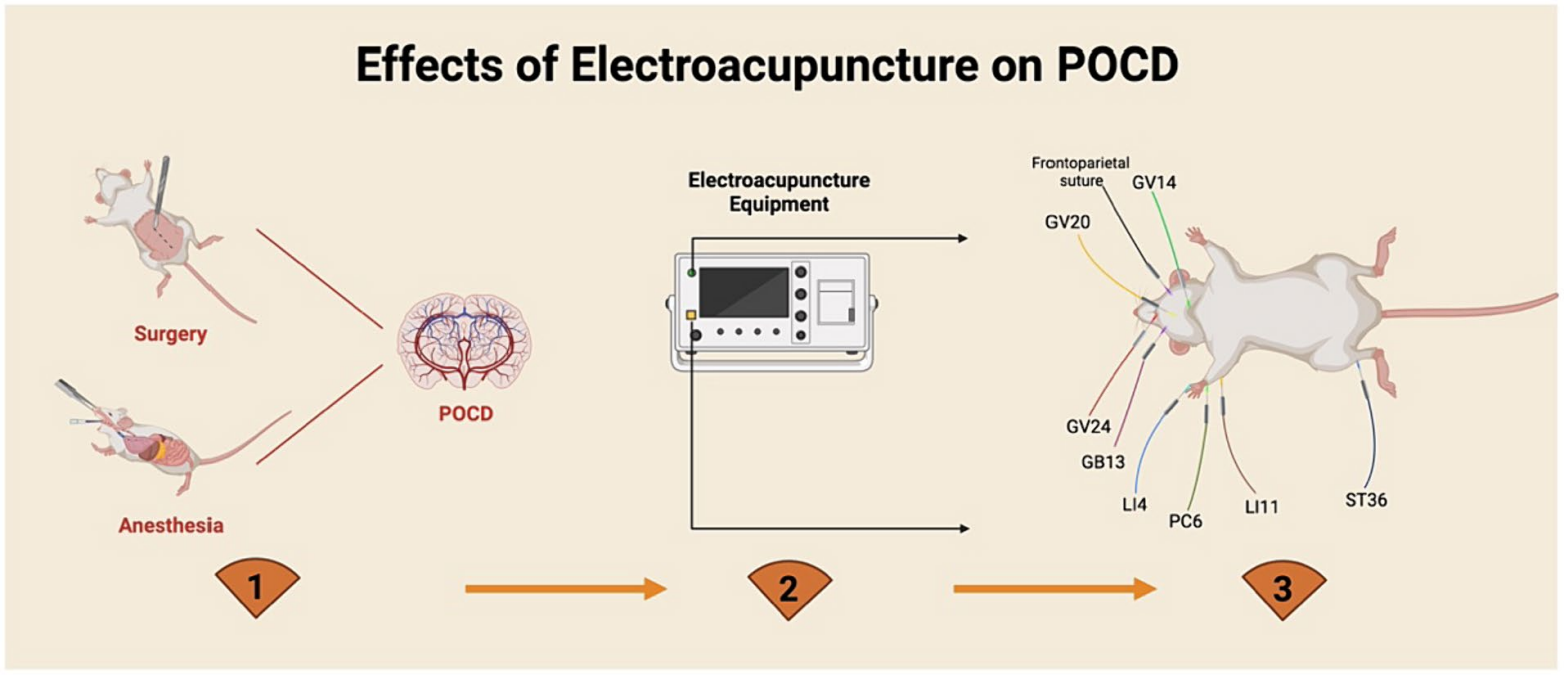
Electroacupuncture intervention for postoperative cognitive dysfunction.

Understanding the mechanisms by which EA affects the development of POCD through randomized controlled trials poses a known challenge. Consequently, animal studies have emerged as the optimal approach to elucidate the biological underpinnings of EA’s impact on POCD. The pursuit of identifying, preventing, and treating POCD remains exploratory due to the absence of standardized diagnostic criteria. Research indicates that EA as a preventive or therapeutic intervention for POCD may elicit various biological responses (Figure 2), such as neuroinflammatory reactions, oxidative stress dysfunction, autophagy disruption, gut microbiota imbalance, and epigenetic modifications.

### 3.1 Neuroinflammation

Surgery and anesthesia may lead to a systemic inflammatory response, potentially causing various reactions that contribute to CNS inflammation. The mechanisms of neuroinflammation in POCD involve the activation of glial cells, disruption of the blood–brain barrier, excessive release of cytokines, recruitment of immune cells, and activation of vagal pathways (Skvarc et al., 2018; Chen et al., 2023). Among these, microglia serve as a crucial point of crosstalk, connecting neuroinflammation with abnormal neuronal function, synaptic injury, and neuronal apoptosis. After neuroinflammation occurs, proinflammatory factors such as TNF-α, IL-1β, and IL-6 induce the transformation of microglia into a toxic proinflammatory phenotype (M1), resulting in microglial morphological dystrophy and dysfunction in oxidative stress (Liu et al., 2023). Indeed, hippocampus-dependent learning and memory are particularly vulnerable to inflammatory damage. A previous preclinical study showed increased IL-1β concentration and microglia activation in the hippocampus (dentate gyrus inner blade, CA1 and CA3) and the predominant cortex of cognitively impaired rats 1 week after surgery (Hovens et al., 2014). Inflammatory factors may indirectly affect neuronal functions essential for learning and memory by modulating intraneuronal pathways. Meanwhile, the decrease in BDNF levels at postoperative weeks 2 and 3 suggests that postoperative neuroinflammation leads to diminished BDNF signaling, decreased neuronal plasticity, and consequently cognitive decline. EA improved cognitive function in POCD rats by elevating serum BDNF levels (Liu et al., 2019). Feng and colleagues demonstrated that EA attenuated postoperative cognitive deficits in aged rats, and this therapeutic effect may be related to the inhibition of hippocampal neuroinflammation via the microglia/TLRs pathway (Feng et al., 2017).

The inflammatory factors released by microglia not only have a direct impact on neurons but can also activate astrocytes. When overactivated, astrocytes alter their morphology, increase the uptake of neurotransmitters, and release inflammatory mediators, which may disrupt the stability of the brain’s environment, interfere with neuronal energy supply, and contribute to or exacerbate cognitive decline. In rats with POCD, EA significantly reduced serum levels of S100 calcium-binding protein B and decreased the number of GFAP-positive astrocytes (Liu et al., 2019). Mast cells (MCs) are effector cells of the innate immune system. They produce a wide array of mediators, including bioamines, cytokines (IL-1, IL-6, TNF-α, transforming growth factor-β) (Sulaiman et al., 2014). MCs can communicate with neurons, microglia, and astrocytes, serving as key players in linking peripheral immune signals with the brain in inflammatory environments (Hendriksen et al., 2017). During stress, disease, or trauma, MCs can become overly activated, impacting the blood–brain barrier, neurons, and glial cells (Skaper et al., 2017). In a model of POCD induced by tibial fracture surgery, EA pre-treatment at the GV20 point inhibited MC degranulation associated with hippocampal α7nAChR activation, downregulated the expression levels of IL-1β, HMGB-1, and TNF-α in serum and the hippocampus, thereby improving cognitive deficits in rats (Zhou et al., 2022).

A large body of evidence suggests the existence of inflammatory pathways mediated by inflammasomes in CNS diseases. Specifically, the NLRP3 inflammasome is present in microglial cells, while neurons contain the NLRP1 and AIM2 inflammasomes, and astrocytes harbor the NLRP2 inflammasome (Wang M et al., 2022; Wang XF et al., 2022). The NLRP3 inflammasome, comprising the NLRP3 protein, pro-caspase-1, and apoptosis-associated speck-like protein containing a CARD (ASC), mediates neuroinflammation that not only directly contributes to the POCD but is also closely associated with high-risk factors for POCD (Li et al., 2022). NLRP3 inflammasome activation occurs in two steps: initiation and activation (Zhang et al., 2023). During the initiation step, cytokine signals stimulate cytokine receptors or TLRs, leading to NF-κB translocation and the promotion of NLRP3 and pro-IL-1β (immature form of inflammatory cytokine) transcription. In the activation step, the NLRP3 inflammasome, upon recognizing a stimulus, facilitates the activation of caspase-1. Activated caspase-1 then converts pro-IL-1β and pro-IL-18 into their active forms, IL-1β and IL-18, respectively. The expression of IL-1β in the brain initiates subsequent inflammatory events, and IL-1β plays a critical role in the development of postoperative infection, preoperative cognitive impairment, and postoperative pain processes (Zhao et al., 2021).

**Figure 3:**
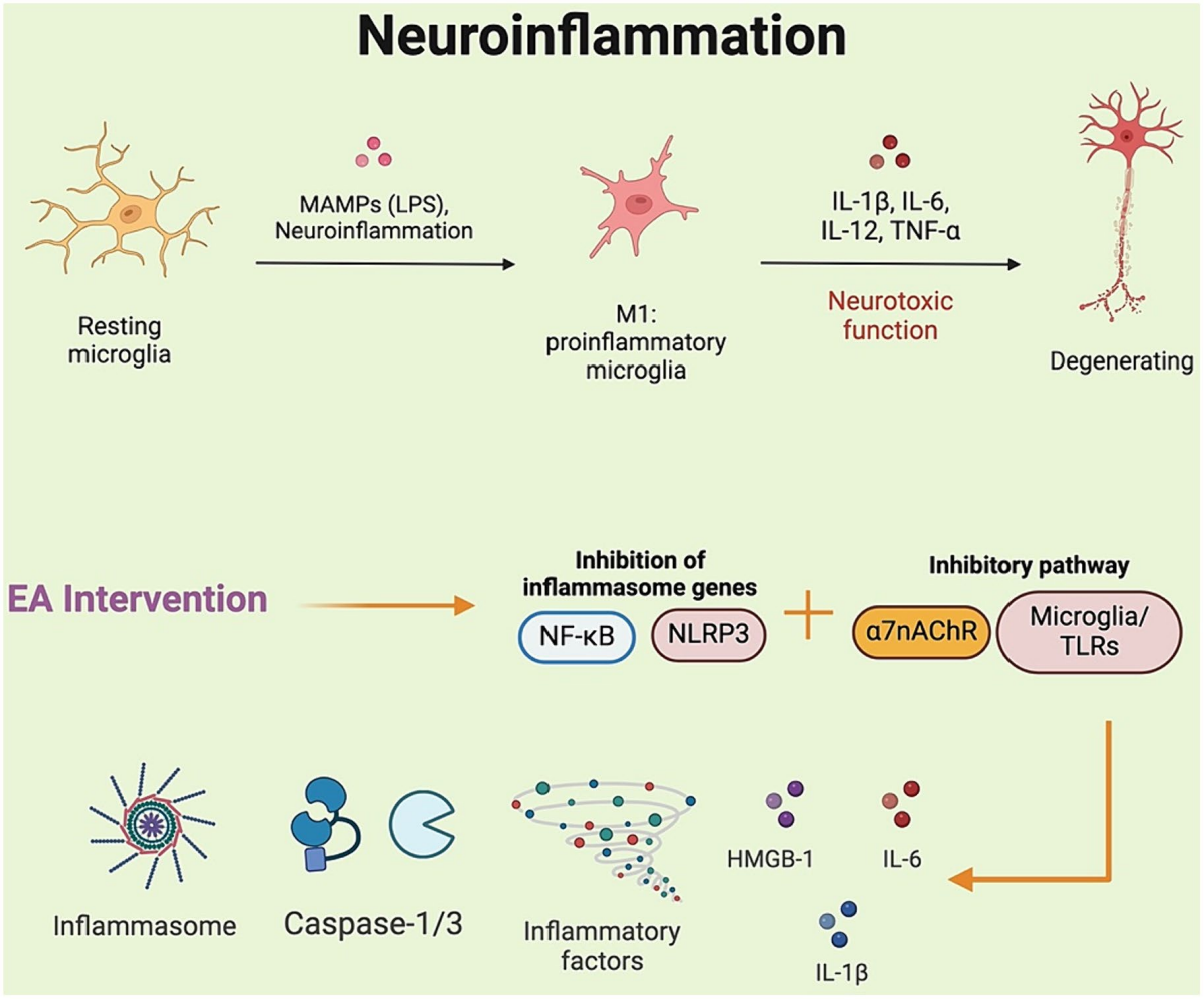
Electroacupuncture and its role in mitigating neuroinflammatory responses in postoperative cognitive dysfunction. NF-κB, nuclear factor-κB.

EA stimulation of ST36 effectively improved motor, spatial learning and memory abilities in mice by reducing microglial activation, decreasing beta-amyloid (Aβ) peptide deposition, and inhibiting the NLRP3 inflammatory response (IL-1β and IL-18 expression) in the hippocampus, thereby attenuating neuroinflammation (Ni et al., 2023). Interestingly, the therapeutic effects of EA were similarly observed in POCD. Sun and colleagues reported that EA’s inhibition of the NLRP3 inflammasome and NF-κB activation alleviated POCD and associated neuroinflammation in aged mice (Sun et al., 2022). Specifically, EA reduced IL-1β and IL-6 expression in the hippocampus and suppressed NF-κB pathway-associated activation of NLRP3 inflammasome, which includes attenuating the recruitment of apoptosis-associated speck-like proteins with a CARD (ASC) and caspase-1. NF-κB is a pivotal upstream regulator of the NLRP3 inflammasome. Although NLRP1, AIM2 inflammasome-mediated ASC-dependent mechanisms are implicated in neuroinflammation, EA did not affect AIM2 expression. Therefore, it may be inferred that the NLRP3 pathway, including its upstream promoters, the NLRP3 inflammasome itself, and activators of the downstream factors (IL-1β, IL-18), could be therapeutic targets for POCD through EA intervention (Figure 3).

### 3.2 Oxidative stress

Elevated levels of reactive oxygen species (ROS) and markers of oxidative damage, along with antioxidant depletion, have been consistently reported following animal surgery (Skvarc et al., 2018). Antioxidant stress is modulated by the activation of antioxidant enzymes and the reduction of pro-oxidant enzymes. Oxidative stress becomes more pronounced in the brain with age. Furthermore, there is an age-dependent decrease in superoxide dismutase (SOD) activity in the brain of aged rats. Oxidative stress compromises mitochondrial function by inducing structural changes, particularly at the mitochondrial respiratory chain complex. Due to the decline in SOD antioxidant defense, mitochondrial function is compromised, potentially leading to protein and DNA damage within mitochondria and providing a possible trajectory for the development of diseases such as AD and POCD. The nuclear factor erythroid 2-related factor 2 (Nrf2) is a key regulator of antioxidant defenses in cells. It is expressed in the hippocampus of the brain, where it regulates the physiological homeostasis of the cellular redox state and the response to stress and inflammation (Li et al., 2023). With age, the Nrf2 system becomes compromised, which is a significant risk factor for nearly all neurological diseases that involve oxidative stress. Deletion of the Nrf2 gene leads to an inability to upregulate the brain’s antioxidant target genes in response to acrylamide. This results in increased activation of microglia, heightened oxidative stress and neuroinflammation, as well as enhanced neurotoxicity in mice (Netto et al., 2018). Activating Nrf2 is one strategy to increase the activity of SOD.

**Figure 4:**
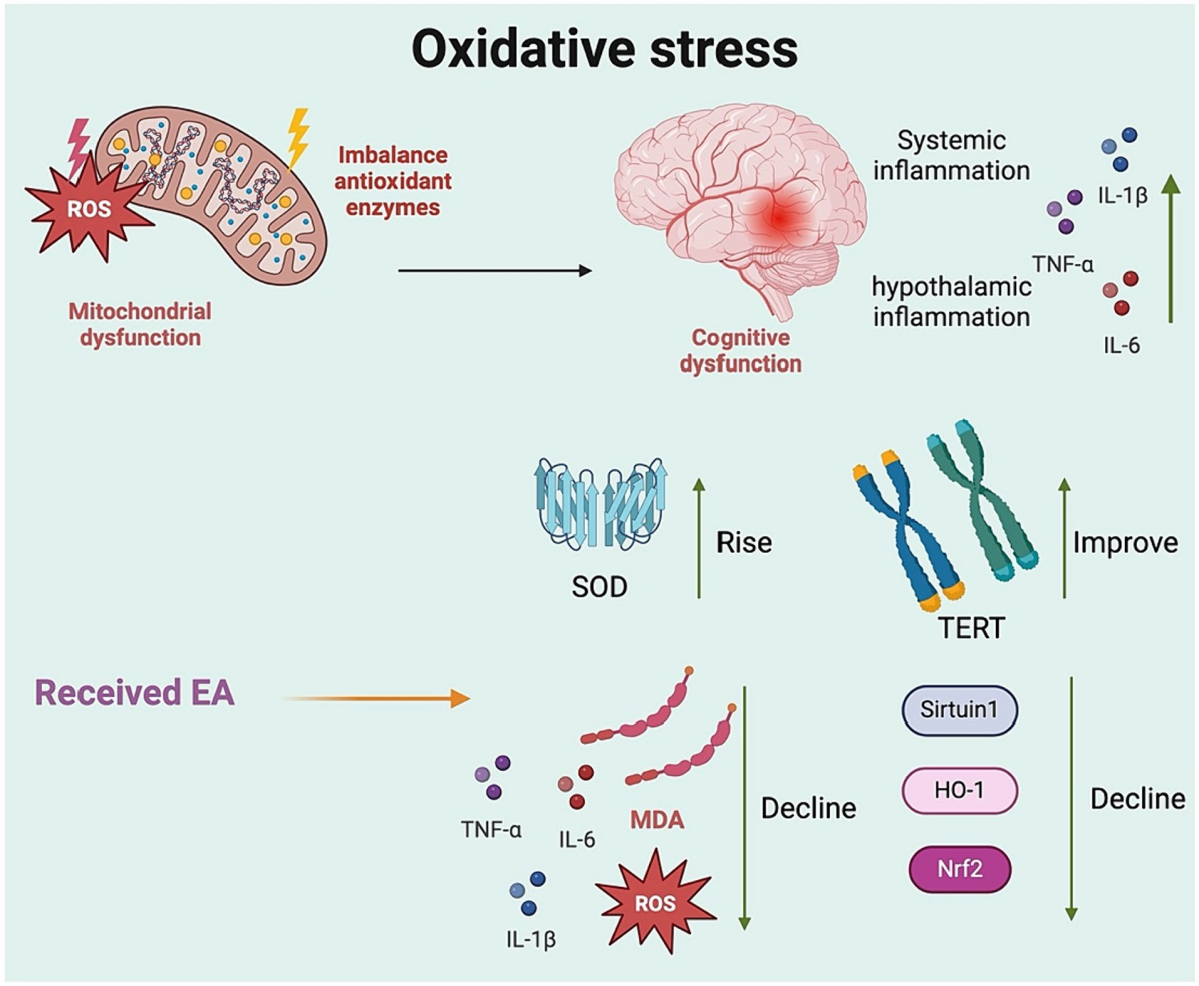
The antioxidative defense mechanism of electroacupuncture in the alleviation of postoperative cognitive dysfunction. SOD, superoxide dismutase; MDA, malondialdehyde; ROS, reactive oxygen species; HO-1, heme oxygenase-1; Nrf2, nuclear factor erythroid 2-related factor 2; AMPK, adenosine monophosphate activated protein kinase.

An experimental model of tibial fracture in rats revealed significant changes in the hippocampus and prefrontal cortex at 24 h and 7 days post-injury. Specifically, aged rats exhibited memory deficits accompanied by increased oxidative stress, mitochondrial dysfunction, and reduced levels of neurotrophic proteins (Netto et al., 2018). Furthermore, various rodent surgical models demonstrated cognitive deficits alongside elevated levels of ROS and proinflammatory mediators in the brain, particularly within the hippocampus, a region crucial for memory formation and learning (Li et al., 2023). EA intervention prevented cognitive deficits in SD rats by enhancing the expression of hippocampal Nrf2, Sirtuin1, and heme oxygenase-1, while concurrently reducing hippocampal ROS levels and inflammatory responses (Tong et al., 2024). In addition, mice that received EA pretreatment showed improved cognitive performance after surgery and enhanced mitochondrial localization of telomerase reverse transcriptase (TERT) (Wang et al., 2023). The underlying mechanisms for these improvements may involve a reduction in oxidative damage and neuroinflammation, a decrease in autophagy dysfunction, and increased telomerase activity and TERT protein expression. Wang et al. (2023) applied EA to the GV20 acupoint in mice, providing electrical stimulation for 10 min daily starting 3 days before surgery. Following EA pretreatment, they observed decreased levels of ROS, MDA, IL-6, and TNF-α, along with fewer Iba1 microglia and increased SOD levels in the hippocampus of aged mice. Elevated oxidative stress is known to impair autophagy, which could play a role in POCD. Notably, EA pretreatment helped preserve TERT function, with this neuroprotective effect being largely ascribed to its anti-inflammatory and antioxidant properties. However, there may be confounding factors affecting this potential mechanism, such as aggregation effects and the natural recovery process of TERT function following surgery. Interestingly, in rats that developed POCD after left hepatic lobectomy, EA intervention significantly suppressed hippocampal oxidative stress levels, reduced astrocyte activation, and lowered serum levels of S-100β and NSE, while increasing serum levels of BDNF and GDBF (Liu et al., 2019). Therefore, both preoperative preconditioning and postoperative management of animals with POCD demonstrated the neuroprotective mechanism of EA stimulation (Figure 4).

### 3.3 Autophagy

Autophagy is an essential and evolutionarily conserved process that maintains cellular homeostasis through the degradation and recycling of cellular contents, misfolded or damaged proteins, and intracellular pathogens (Awi et al., 2021). It also plays a role in the elimination of Aβ aggregates and neuronal tau. A defective autophagy response has been shown to lead to the accumulation of Aβ and abnormal tau phosphorylation, exacerbating cognitive impairments in various neurodegenerative diseases (Zhang et al., 2021). Notably, the role of autophagy in POCD is controversial. Some studies have reported a protective role for autophagy in POCD (Yang et al., 2020). Suppression of autophagy is accompanied by severe α-synuclein aggregation and overproduction of harmful oligomers in the hippocampus, leading to neurotransmitter imbalance and hippocampus-dependent cognitive deficits. Studies suggest that α-synuclein or its oligomers may contribute to the development of POCD. Moreover, overexpression of AMPKα1 demonstrated a significant neuroprotective effect against α-synuclein toxicity *in vivo*. This overexpression led to a marked elevation in the LC3-II/LC3-I ratio and Beclin1 protein levels, along with a substantial reduction in p62 protein levels in the hippocampus of POCD rats. These findings suggest that overexpression of AMPKα1 can activate AMPK-Sirt1 pathway and autophagy signaling, thereby preventing learning and memory deficits in POCD rats (Yan et al., 2019).

Conversely, other studies have reported opposite results, indicating that interventions like rapamycin—an mTOR inhibitor—can inhibit the mTOR signaling pathway and activate autophagy, resulting in improved memory and cognitive function following surgery. Rapamycin reduces tau protein phosphorylation and increases levels of synaptophysin, BDNF, and atg5 expression in mice. Furthermore, EA has been observed to enhance autophagy initiation, autophagosome biogenesis, mitochondrial autophagy, and autophagic flux/substrate degradation in certain brain regions (Huang et al., 2023).

**Figure 5:**
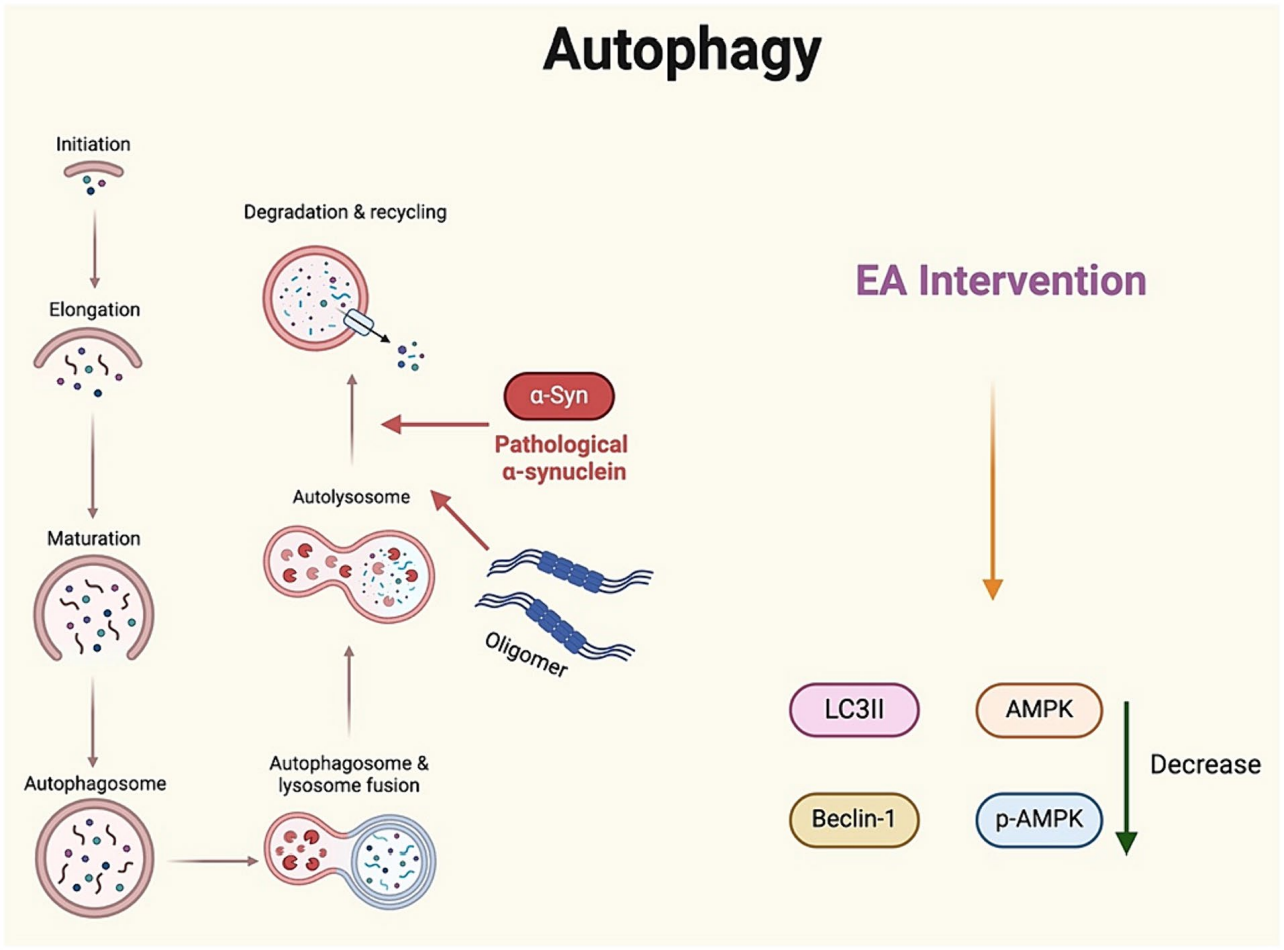
Regulation of autophagy by electroacupuncture: implications for postoperative cognitive dysfunction treatment. α-Synα, α-synuclein; IL, interleukin.

A study demonstrated that EA may confer a protective effect against POCD by inhibiting excessive autophagy in rat hippocampal tissue (Niu et al., 2022). In the study, rats received EA pre-stimulation at GV14 and GV20 for 30 min daily over a period of 5 days. Following surgery, EA treatment reduced the expression of LC3II, Beclin-1, AMPK, and p-AMPK, effectively inhibiting autophagy in the hippocampal tissues of aged POCD rats. LC3II and Beclin-1 are well-established autophagy markers. Meanwhile, they measured the formation of autophagic vesicles in rat hippocampal tissues found that the number of autophagic vesicles was higher after surgery. After pretreatment with EA, the number of autophagic vesicles was significantly lower. Although EA intervention may become a compelling approach to manage POCD, a deeper understanding of autophagy in the context of the POCD process is still required to comprehend the mechanisms of this complexly regulated, highly dynamic cellular event (Figure 5).

### 3.4 Microbiota-gut-brain axis

There is a close connection between the gut microbiota and the CNS through neural, immune, endocrine, and metabolic pathways. Disturbances in the gut microbiota leads to neuroinflammation and promote microglia activation and pathological changes in brain tissue (Huang et al., 2022). The gut microbiota may contribute to POCD through the gut-brain axis. One possible explanation is that surgical trauma causes gut microbial dysbiosis, altering metabolites that affect POCD. Cao et al. (2024) provided evidence supporting a potential causal relationship between genetically predicted gut microbiota and metabolites and cognitive performance, based on large-scale GWAS summary data (Cao et al., 2024). Additionally, randomized controlled studies have demonstrated the potential of specific probiotics (*Bifidobacterium longum*) to ameliorate cognitive impairment (Kim et al., 2021).

**Figure 6:**
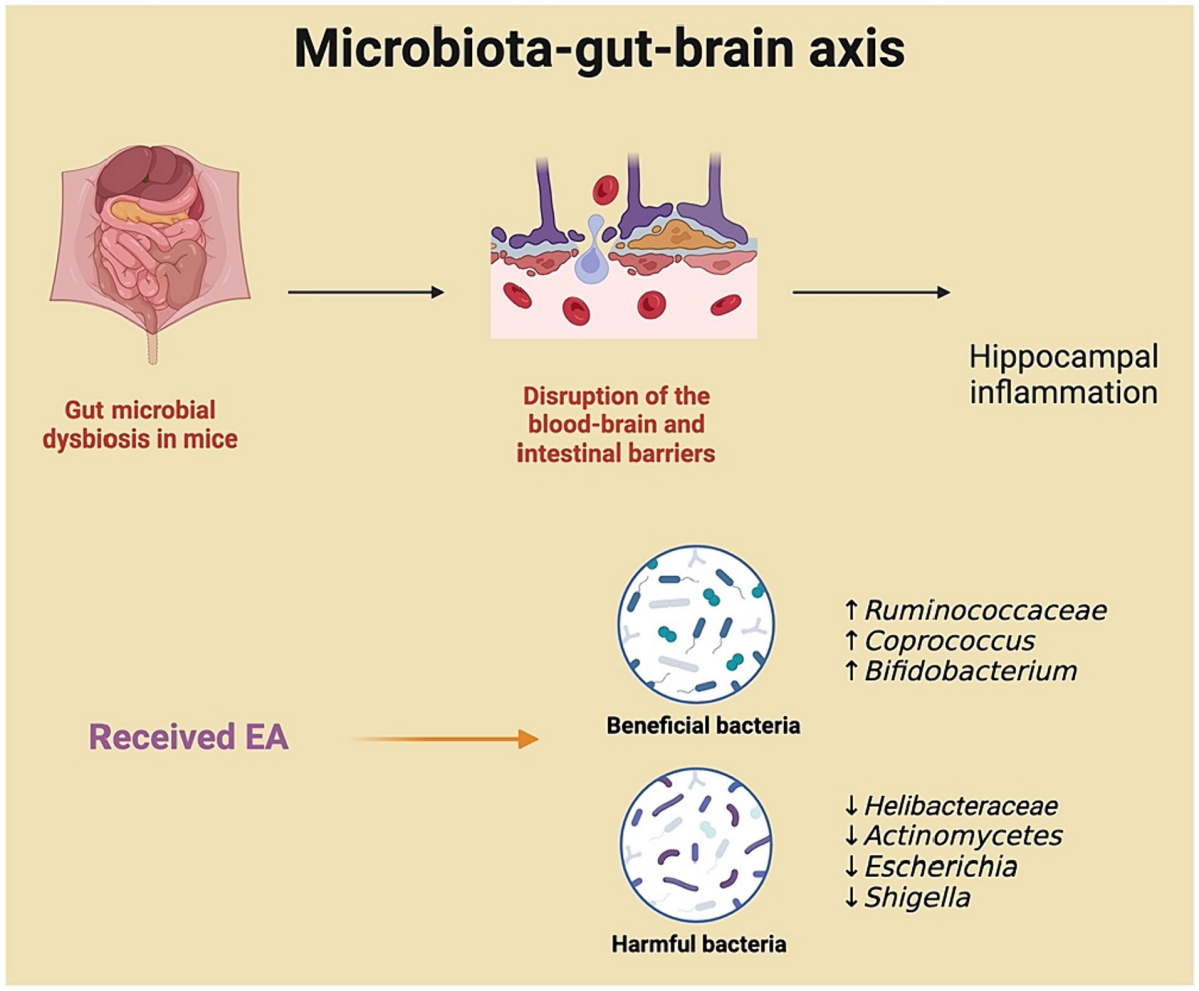
Electroacupuncture’s influence on the microbiota-gut-brain axis in preventing postoperative cognitive dysfunction.

A recent animal study has found that alterations in the gut microbiota composition coincide with changes in the permeability of critical barriers, such as the blood–brain and intestinal barriers. This permeability allows inflammatory mediators to enter the bloodstream, ultimately exacerbating hippocampal inflammation. The study demonstrated improved cognitive function, which had been impaired by abdominal surgery, through EA intervention at four acupoints (ST36, LI11, GV20, GV14) (Zhu et al., 2023). Specifically, EA decreased the postoperative abundance of *Helibacteraceae, Actinomycetes, Escherichia/Shigella*, while it increased the abundance of short-chain fatty acids (SCFA)-producing bacteria like *Coprococcus* and *Ruminococcaceae*. SCFAs play a protective role in maintaining the integrity of the gut and the blood–brain barrier (BBB), safeguarding against harmful gut microbiota that could compromise brain function. In addition, SCFAs were shown to reverse signs of microglial immaturity and malformation in germ-free mice (Kim et al., 2021). EA also mitigated evidence of BBB damage in mice with POCD, improved the integrity of endothelial cell tight junctions, and reduced postoperative astrocyte activation. The finding that EA pretreatment attenuates POCD, as explained through the lens of gut microbiota and derived metabolites, may offer a new potential mechanism for managing POCD (Figure 6).

### 3.5 Epigenetic modification

Epigenetics primarily encompasses DNA methylation, histone modifications, non-coding RNA (ncRNA), and RNA methylation (Choo, 2011). The mechanisms of DNA methylation and histone modification are implicated in transcriptional events, while ncRNA and RNA methylation influence post-transcriptional gene regulation and protein synthesis (Wu et al., 2023). Regulatory RNA networks, along with these epigenetic mechanisms, are extensively linked to transcriptional alterations in genes that are crucial for long-term memory and cognitive processes within the CNS. MiRNAs, small ncRNAs (~22 nt) that bind to complementary sequences at the 3’-UTR of protein-coding mRNAs, play crucial roles in neuronal development, neuroplasticity, and cognition. A recent experimental study revealed that the overexpression of miR-146a ameliorated learning and memory deficits related to the hippocampus in mice with POCD. This effect was accompanied by decreased expression of the IRAK1/TRAF6/NF-κB signaling pathway and downregulation of microglia activation in the hippocampus (Chen L et al., 2019).

**Figure 7:**
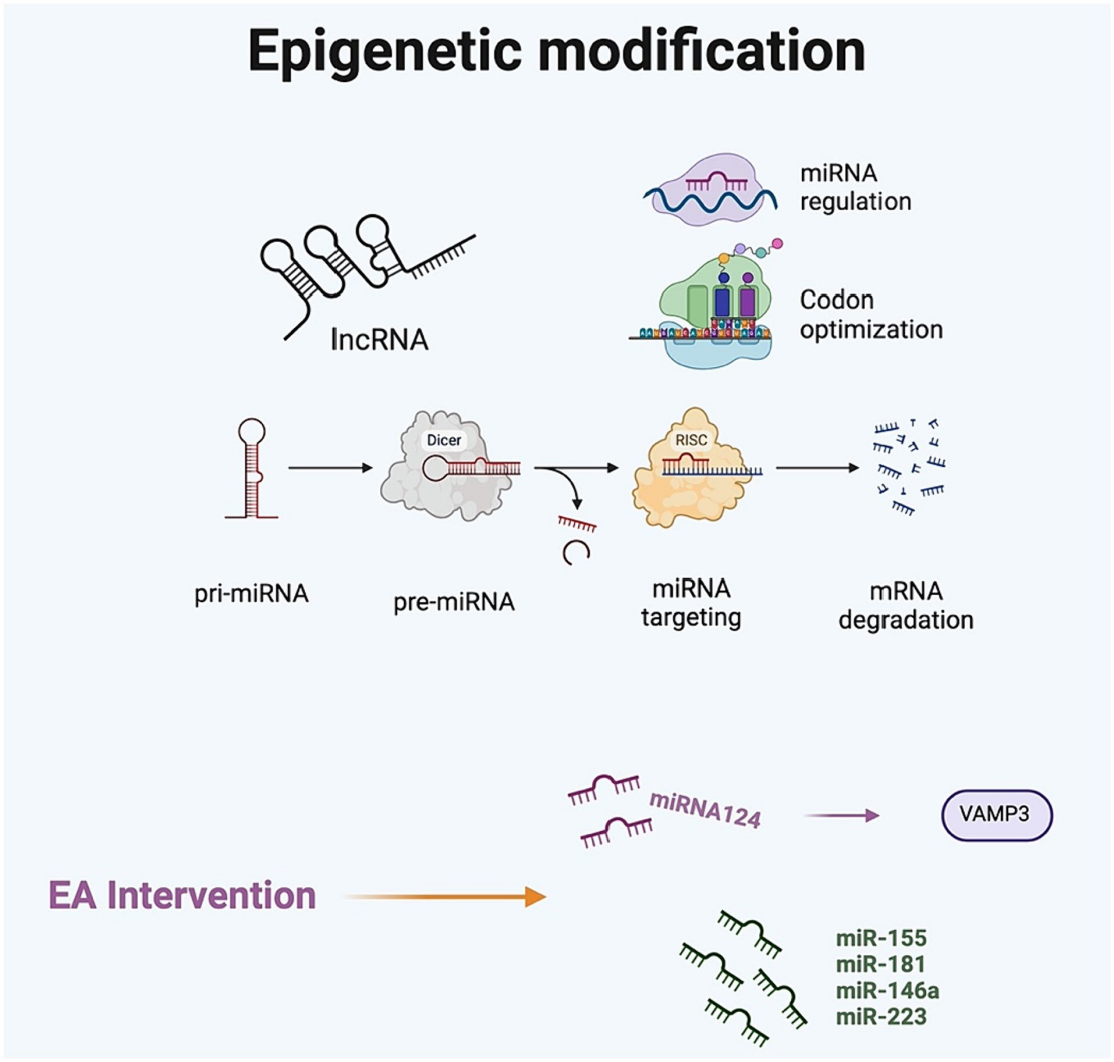
The impact of electroacupuncture on epigenetic modifications in postoperative cognitive dysfunction.

miR-124 is one of the most abundant microRNAs in the brain, playing a crucial role in regulating neurological development and maintaining microglia in a resting state. Abnormal expression of miR-124 has been implicated in the onset and progression of POCD, leading to microglia activation and the release of inflammatory mediators under stress. Animal studies have shown that EA can inhibit microglia activation (Chen et al., 2016). Chen Y et al. (2019) utilized EA as a therapeutic intervention to investigate that involvement of miR-124 and its downstream pathway VAMP3 in the development of POCD. In their study, POCD-model rats received EA stimulation at the bilateral ST36 acupoints. The treatment markedly reduced microglial activation in the hypothalamus and hippocampus and increased miR-124 expression within these cells. Interestingly, they also observed alterations in neurimmiR expression, including miR-155, miR-181, miR-146a, and miR-223. Notably, while the potential mechanisms of EA treatment are linked with novel therapeutic targets for miRNAs and offer promise as biomarkers for POCD, the identification of individualized therapeutic regimens for each patient is crucial. Moreover, further research is necessary to ascertain their utility in clinical settings (Figure 7).

## 4 Discussion

Emerging evidence from rodent studies underscores the neuroprotective potential of EA in alleviating POCD, a complex neurocognitive syndrome that significantly impacts cognitive health in the elderly post-surgery. This review delves into the multifaceted biological mechanisms through which EA operates, including anti-neuroinflammation, enhancement of antioxidative defenses, modulation of autophagy, restoration of gut microbiota balance, and influence on epigenetic modifications. While preclinical studies support the beneficial effects of EA, translating these mechanisms into clinical efficacy remains in its infancy. This discussion aims to explore the implications of our findings, the inherent limitations of current research, and provide direction for future exploration.

Firstly, neuroinflammation is a pivotal factor in the onset of POCD, with EA showing significant anti-inflammatory effects by reducing the production of inflammatory mediators and the activation of microglia. This anti-inflammatory action could be mediated by modulating NLRP3 inflammasome activation and reducing pro-inflammatory cytokine release. Notably, there’s a close relationship between neuroinflammation and oxidative stress, as inflammatory processes can generate a substantial amount of ROS, thereby exacerbating oxidative stress. Thus, the anti-inflammatory action of EA might indirectly promote neuroprotection by alleviating oxidative stress. The role of the gut-brain axis is also undeniable, with studies indicating that EA can improve the composition of the gut microbiota, thereby alleviating POCD through anti-inflammatory and antioxidative mechanisms. Additionally, epigenetic modifications, as crucial regulators of gene expression, have profound impacts on cognitive functions and neuroprotection. EA’s influence on specific miRNA expressions could modulate signaling pathways related to inflammation, oxidative stress, and autophagy, offering a novel perspective for POCD treatment.

It must be emphasized that much of our understanding of POCD and its mechanisms stems from preclinical research using animal and cell models. Moreover, cohort studies exploring EA’s treatment of POCD are scarce, highlighting the necessity of integrating these research findings with clinical practice. This raises concerns regarding the reliability of these findings in reflecting the actual clinical condition in human patients. To convert these insights into therapeutic interventions, it is vital to tailor studies involving human subjects to adopt a holistic methodology, which includes variations in acupuncture techniques, intensities, and acupuncture points. Such research should adhere to stringent standards, pinpointing certain stages of treatment (before or after surgery) and employing diverse methods to confirm the efficacy of EA. Future research should aim to validate the efficacy of EA in improving POCD and its underlying mechanisms through longitudinal cohort studies, bridging the gap between preclinical research and clinical application.

Presently, a notable gap in research is the variability in experimental protocols and design. For instance, cognitive assessments in animal studies have largely concentrated on the hippocampus. However, many other brain regions, including the cerebellum and thalamus, also play crucial roles in the formation and processing of learning and memory. This gap highlights the urgent need to extend POCD research beyond the hippocampus to include these broader brain areas involved in cognitive functions.

While we have integrated a range of mechanisms through which EA impacts POCD, there is a notable lack of content regarding the mechanisms of innate and adaptive immune cell infiltration. Future studies should consider including aspects such as immune cells in POCD research. Additionally, neuroimaging techniques offer detailed insights into brain function and structure at the molecular, cellular, and systemic levels. Researchers can monitor changes in brain function and structure in real time within animal models, understanding how brain networks are affected during disease processes. Investigating the effects of EA through rs-fMRI in animal models can provide a theoretical basis and empirical evidence for its clinical application. Understanding the impact of EA on brain functional connectivity can help optimize clinical treatment protocols for EA, such as selecting the most effective stimulation parameters and acupoints. Neuroimaging technologies like rs-fMRI represent a potential direction for future research.

## 5 Conclusions and perspective

In summary, our review highlights the broad but underexplored potential of EA to improve POCD through various biological mechanisms. While preclinical models offer valuable insights, overcoming current limitations and exploring new research avenues are necessary to translate these findings into effective clinical interventions. Future studies should aim to expand the scope of research beyond the hippocampus, employ innovative methods like rs-fMRI, and conduct longitudinal cohort studies to determine the clinical efficacy and safety of EA treatment for POCD. By further understanding the mechanisms of action of EA and optimizing its application in clinical settings, we may find new therapeutic strategies to alleviate POCD and improve patients’ quality of life.

## Data availability statement

The original contributions presented in the study are included in the article/supplementary material, further inquiries can be directed to the corresponding author.

## Author contributions

WZh: Conceptualization, Investigation, Methodology, Writing – original draft, Writing – review & editing. WZo: Conceptualization, Supervision, Validation, Visualization, Writing – original draft, Writing – review & editing.
